# A Genomic Perspective on the Evolutionary Diversity of the Plant Cell Wall

**DOI:** 10.3390/plants9091195

**Published:** 2020-09-12

**Authors:** Ryusuke Yokoyama

**Affiliations:** Graduate School of Life Sciences, Tohoku University, Sendai 980-8578, Japan; ryusuke.yokoyama.d6@tohoku.ac.jp; Tel.: +81-022-795-6702

**Keywords:** plant cell wall, dynamic structure, polysaccharide, cell wall enzyme, gene family, genome, evolution, comparative analysis

## Abstract

The plant cell wall is a complex and dynamic structure composed of numerous different molecules that play multiple roles in all aspects of plant life. Currently, a new frontier in biotechnology is opening up, which is providing new insights into the structural and functional diversity of cell walls, and is thus serving to re-emphasize the significance of cell wall divergence in the evolutionary history of plant species. The ever-increasing availability of plant genome datasets will thus provide an invaluable basis for enhancing our knowledge regarding the diversity of cell walls among different plant species. In this review, as an example of a comparative genomics approach, I examine the diverse patterns of cell wall gene families among 100 species of green plants, and illustrate the evident benefits of using genome databases for studying cell wall divergence. Given that the growth and development of all types of plant cells are intimately associated with cell wall dynamics, gaining a further understanding of the functional diversity of cell walls in relation to diverse biological events will make significant contributions to a broad range of plant sciences.

## 1. Introduction

A key distinguishing feature of plants is that individual cells are surrounded by a cell wall, which confers mechanical strength that contributes to the maintenance of cell shape and provides sufficient flexibility to facilitate cell expansion [1]. Consequently, the cell wall plays crucial roles in multiple aspects of plant development, growth, and differentiation. The basic framework of the primary cell wall is mainly composed of cellulose microfibrils and hemicellulosic polysaccharides, such as xyloglucan, and embedded in a complex matrix of pectins [2,3,4]. It has previously been proposed that xyloglucan interacts with two or more cellulose microfibrils to form a tether between cellulose microfibrils [5,6,7]. However, recent experimental investigations, along with the development of new technologies, have led to the proposal of a new model, in which the xyloglucan is closely intertwined with cellulose microfibrils at limited sites and mechanically contributes to the network structure [8,9,10]. The entire cell wall is a more complex structure, comprising a diverse range of polysaccharides, highly glycosylated proteins, and phenolic compounds, and its composition is differentially controlled according to cell type and in relation to different stages of growth and development [11,12]. Additionally, some types of cells, such as xylem cells, develop secondary cell walls, which are characterized by an abundance cellulose and xylan, and further reinforced with lignin [13,14,15,16,17].

Recent advances in technology have provided snapshots of plant cell walls from multiple viewpoints. Atomic force microscopy (AFM) has made it possible to directly image the wall architecture at high resolutions, particularly the cellulose microfibrils, and to visualize the alteration in microfibril connectivity involved in wall loosening [18,19,20]. Important insights have also been gained regarding the precise polysaccharide conformation and interactions that underlie cell wall assembly, based on solid-state nuclear magnetic resonance (NMR) studies [21,22]. Additionally, the application of monoclonal antibodies (mAbs) raised against wall polysaccharides has been developed as a powerful tool for examining the precise localization of the polysaccharides and wall microstructures via a wide range of experimental techniques, including immunohistochemical analysis and carbohydrate microarray binding profiles [23,24,25]. Several mAbs can also be used to characterize the different status of wall polysaccharides. For example, LM19 and LM20 are well known to have differing specificities in relation to the methyl-esterification of homogalacturonan (HG), which has roles relating to cell growth and development [26]. Furthermore, the combination of other new technologies has given more powerful tools for analyzing the wall microstructures. The combination of xyloglucan-directed mAbs and high-resolution imaging by field emission scanning electron microscopy has provided insights into xyloglucan conformation and its interactions with cellulose, which are essential features contributing to the basic framework of the plant cell wall [27].

Real-time imaging of cell wall polysaccharides based on chemical staining is also one of the most powerful techniques for monitoring the dynamics of wall microstructures. For example, staining with Pontamine Fast Scarlet 4B, a dye that fluoresces in the presence of cellulose, facilitates the imaging of cellulose dynamics, and has revealed that cellulose bundles rotate in a transverse to longitudinal direction during cell expansion [28,29]. Additionally, we recently developed an imaging technique that can be used to quantitatively evaluate the network of cellulose microfibrils [30,31]. By combining this quantitative imaging technique with a high-yield cell-wall regeneration procedure, we successfully quantified the total length, mean intensity, skewness of intensity distribution, and coefficient of variation of regenerating cellulose microfibrils in protoplasts derived from *Arabidopsis* leaf mesophyll cells [31]. Furthermore, by adopting a quantitative imaging approach using a xyloglucan-deficient *xxt1/xxt2* mutant of *Arabidopsis thaliana*, we showed that the absence of xyloglucans has almost no influence on either the structure of the cellulose microfibril network or protoplast stability in regenerating protoplasts, thereby indicating that xyloglucan does not directly contribute to the initial assembly of the cellulose network or the mechanical strength of the cell wall of protoplasts [32]. Given that xyloglucan plays an important role in wall loosening, these observations also indicate that the roles of xyloglucan in the initial assembly of cell walls are distinct from those in the cell wall of expanding cells.

## 2. Cell Wall Diversity and Plant Evolution

In addition to facilitating high-resolution imaging of the cell wall structure, advanced technologies have also revealed that there exists a wide range of structurally and functionally distinct cell walls among different plant species, as well as between discrete developmental stages and cell types within a single plant species. 

Unicellular green algae in the division Chlorophyta have a relatively fragile cell wall, or lack a structured cell wall. However, some unicellular green algal species, such as *C. reinhardtii*, have a glycoprotein-layer structure similar in composition to those of land plant species [33]. Additionally, quite a few members of cell wall gene families have been found in unicellular green algae genome sequences [34,35]. For example, the *C. subellipsoidea* C-169 genome reveals cellulose synthase-like domains, although not orthologous to the cellulose synthases and hemicellulose synthases of land plant species [36]. Furthermore, Charophyte green algae have a highly similar cell wall structure to that of land plants, and share many cell wall components with land plants. This is also supported by the presence of genes involved in biosynthesis of the major polysaccharides found in land plants [37]. On the other hand, recent studies have also provided insight into marked differences in cell wall structure and composition between green algae and land plants. For example, although the macromolecular pectic network plays multiple roles in the dynamic structure and ionic environment of the plant cell wall, some pectic network domains, such as arabinans and rhamnogalacturonan I (RG-I), have been found to be less abundant in green algae [38,39,40]. Additionally, the cross-linking of rhamnogalacturonan II (RG-II) via a borate diester, which is essential for the structural organization of the cell wall in angiosperms, has not been found in either bryophytes or charophytes [41]. In contrast, homogalacturonan (HG), which forms complexes with Ca^2+^, has been characterized as a major component of both land plants and Zygnematophyceaen green algae, the closest relatives of land plants [42]. This diversity in cell wall characteristics may be closely related to certain prerequisites for terrestrial survival, or an adaption to terrestrial habitats that has developed during the evolution of land plants. 

Cell wall diversity is also conspicuous with respect to land plant linkages. In many species of terrestrial plants, the primary cell wall is composed mainly of a cellulose–xyloglucan framework embedded in a macromolecular pectic network. However, in commelinid monocotyledons, which include cereals such as rice (*Oryza sativa*), the primary cell walls contain only small amounts of xyloglucan, and instead contain glucuronoarabinoxylan and β1,3:β1,4 mixed glucans as the predominant glycans that interact with the cellulose microfibrils [43,44,45]. This type of cell wall also contains less pectin and higher amounts of hydroxycinnamates, such as ferulate and *p*-coumarate, which form extensive interconnecting networks [46,47]. For example, the residues of ferulic acid are esterified to the arabinosyl side chains of arabinoxylans, and oxidative coupling of the ferulate side chains leads to the formation of cross-links between the arabinoxylans, thereby generating arabinoxylan networks [48,49]. Although the precise roles of polysaccharides and their cross-linkages remain to be elucidated, commelinid monocotyledons may have developed unique cell wall network structures to adapt to the environmental conditions in their respective habitats [50].

The diversity of the cell wall structure and composition provides compelling evidence as to the significant role that this cellular component has played in the evolutionary history of plant species [51,52]. However, our current knowledge of the contribution of the cell wall to plant evolution is still relatively limited. The challenge now is to gain a more comprehensive understanding of the functional diversity of the cell wall in relation to diverse biological events in different species.

## 3. Recent Updates and Developments in the Databases of Plant Genomes and Cell Wall Genes

The rapid development of DNA sequencing technologies has provided useful resources that will contribute to enhancing our understanding of cell walls. A large number of cell wall datasets have accumulated in general public databases and plant-specific public databases, such as Phytozome (https://phytozome-next.jgi.doe.gov). Phytozome is one of the most comprehensive plant genome databases and provides access to the sequences and functional annotations of complete plant genomes, including those of land plants and algae sequenced at the Joint Genome Institute and elsewhere [53]. Additionally, the 1000 plant transcriptomes initiative (1KP) has generated sequence resources for over 1000 plant species, including all of the major lineages of green plants [54], and a further project to sequence 10,000 plant species genomes is now in progress [55]. The availability of large sets of plant genomes provides an opportunity for meaningful comparisons of genes in widely divergent plant species, thereby enabling us to gain a broader perspective on the evolution and functional diversification of cell wall genes. Using phylogenomic synteny analysis, it is possible to identify orthologous genes among different plant species and predict those cell wall genes that have evolved uniquely in some plant lineages [56]. Comparative approaches to characterizing diversity patterns among gene family members are also potentially useful with respect to resolving the relatedness between cell wall enzymes in the metabolic pathways [57].

The structural and functional diversity of complex carbohydrates in the cell wall are controlled by an array of enzymes, including glycosyltransferases (GTs), glycosylhydrolases (GHs), polysaccharide lyases (PLs), and carbohydrate esterases (CEs), designated as carbohydrate-active enzymes (CAZymes) in the Carbohydrate-Active enZymes database (CAZy; http://www.cazy.org) [58]. CAZy is a particularly useful resource for the classification of plant cell wall enzymes, which facilitates the prediction of a broad category of carbohydrate substrates based on the assignment to a family, although it is difficult to establish the precise specificity [59]. The CAZyme classification system has been widely accepted by plant researchers, and the number of CAZy families has expanded following the identification and characterization of novel plant CAZymes, for example, GT106, in different plant species [60]. With an increasing number of plant genomes being sequenced, a large number of sequences have been assigned to CAZy families. The identification of all CAZymes encoded by a given plant genome will provide insights into the nature of complex cell wall carbohydrates in that plant species.

## 4. Comparative Plant Genome Analysis of Plants with a View toward Characterizing of Cell Wall Diversity

### 4.1. Comparative Analysis of the Cell Wall Gene Families among 100 Plant Species

**Figure 1:**
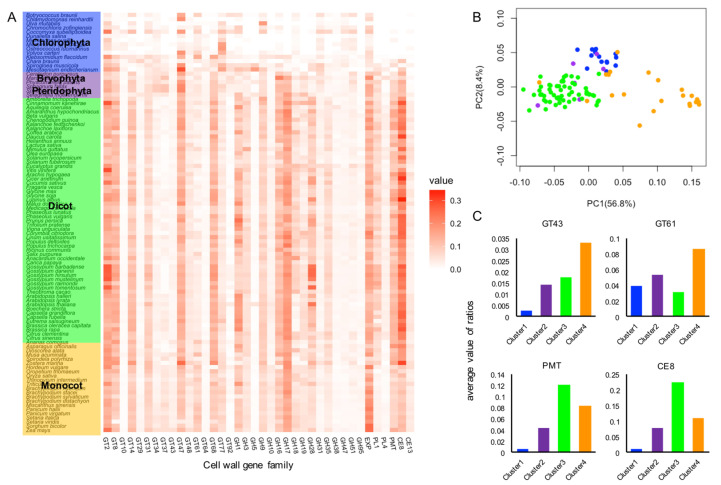
Comparative analysis of cell wall family genes in 100 plant species. (**A**) A heatmap diagram of cell wall family genes in 100 plant species. The heatmap represents the ratio of the number of members in each family to the total number of protein-coding genes (Table S1). The datasets were obtained from public databases (https://phytozome-next.jgi.doe.gov; https://bioinformatics.psb.ugent.be/orcae/) [53,63]. Additionally, the sequences for *K flaccidum*, *S. muscicola* and *M. endlicherianum*, were collected from the *K. nitens* NIES-2285 genome project and the China National GeneBank (CNGB) Nucleotide Sequence Archive (CNSA: http://db.cngb.org/cnsa; accession number CNP0000746), respectively [64,65]. The details of the selected plant species including its database version are also described in Table S2. The criteria used in previous studies were adopted for identifying the members of each family [61,62], and gene family names are defined in the Carbohydrate-Active enZymes database (CAZy), except for expansin and pectin methyltransferase (PMT) (http://www.cazy.org) (Table 1). (**B**) Two-dimensional principal component analysis (PCA) score plots of plant species using the pattern of the cell wall families. PCA was carried out using the function prcomp in R version 3.6.3. Each point corresponds to the member of Chlorophyta (blue), Bryophyta or Pteridophyta (purple), dicots (green), and monocots (orange). (**C**) Average values of the ratio of the number of the family genes in the clusters identified by k-means clustering analysis. A partition of plant species was performed using the k-means clustering method implemented in scikit-learn python package, with the number of partitions set to four (Table S3). The cell wall gene families making significant contributions for each cluster were identified by comparing the average value of the ratio of the family genes within each cluster. Xylosyltransfearse (GT43), arabinosyltransferase (GT61), PMT, and pectin methylesterase (CE8) are shown as the representative families for clusters 4 and 3, respectively.

As an example of a phylogenomic approach, I herein describe the diversity patterns of cell wall gene families in 100 species of green plants. Plant genome datasets were obtained from public databases (https://phytozome-next.jgi.doe.gov; https://bioinformatics.psb.ugent.be/orcae/; http://www.plantmorphogenesis.bio.titech.ac.jp/~algae_genome_project/klebsormidium/index.html; http://db.cngb.org/cnsa). I examined the available annotations of the genome sequences to identify plant cell wall genes, and classified a total of 85,803 genes into 38 cell wall gene families, including GTs, GHs, PLs, and CEs, and two additional cell wall gene families, expansin (EXP) and pectin methyltransferase (PMT) (Table 1). With the exception of the EXP and PMT family, the cell wall gene family names are defined according to the CAZyme repertoires (http://www.cazy.org). The criterion for classification was described in greater detail in a previous report [61,62]. I found that the total numbers of cell wall genes were generally high in land plants, which contrasts with the total numbers identified in green algae (Figure S1). The total counts of cell wall genes in individual plant species ranged from 45 in *Micromonas pusilla* to 2911 in *Thinopyrum intermedium*. To take into account the fact that variations in gene numbers may simply reflect differences in genome size, I further assessed the ratio of the number of members in each gene family to the total number of protein-coding genes and visualized differences in the ratios by generating a heatmap (Figure 1A). 

**Figure 2:**
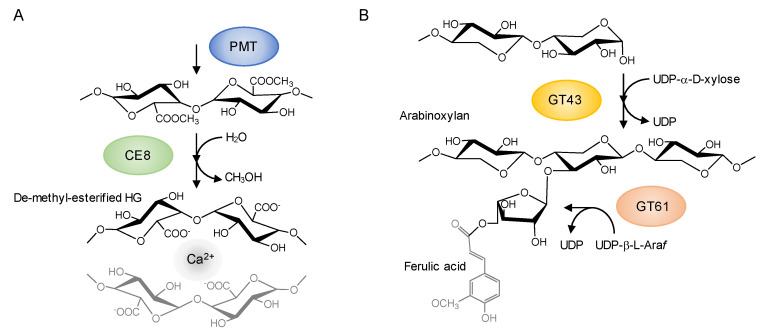
The molecular processes involved in GT43, GT61, PMT and CE8 family enzymes. (**A**) Homogalacturonan (HG) modification processes by PMT and pectin methylesterase (PME) in CE8. The de-methylesterification of HG regulated by PME leads to the interaction with Ca^2+^ ions between the unesterified carboxyl groups of the galacturonosyl residues of two HG chains. (**B**) The biosynthesis processes of arabinoxylan by xylosyltransfearse (GT43) and arabinosyltransferase (GT61) [57].

The results revealed certain approximate trends in the diversity patterns of the family members. For example, almost no members of the CE8, CE13, and PMT families are present in derived green algae before the branching of the Charophyceae, although relatively large numbers of these family members are found in land plant species. PMT catalyzes the methyl-esterification of homogalacturonan (HG) in the medial Golgi, and pectin methylesterase (PME) in CE8 catalyzes the de-esterification of methyl-esterified HGs in muro, followed by the formation of intermolecular Ca^2+^ bonds, thereby forming a rigid gel (Figure 2A) [66]. The CE13 family genes encode pectin acetylesterases (PAE), which can cleave the acetylester bond from pectic polysaccharides, such as HG and rhamnogalacturonan I (RG-I), and thereby modulate the degree of acetylation of pectic polysaccharides. The regulation of pectin acetylation by PAE is considered to be involved in controlling the mechanical properties of the cell wall [67]. Additionally, it should be noted that members of PL1 and GH28, including pectinase-encoding genes, are not found in these algal lineages. These results indicate the possibility that pectin modification involved in the extensive expansion of these gene families is linked to the structural and functional characteristics of the cell wall for adaptations to terrestrial habitats [68,69]. In this regard, it is of particularly interest to note that a large number of these family members have been found in *Penium margaritaceum*, an archetype of the Zygnematophyceae, the closest relatives of land plants [70,71,72]. The plant cell wall has long been considered a key factor associated with adaptations to terrestrial habitats and, accordingly, more detailed comparative studies of early diverging land plant lineages and their sister algal lineages may potentially contribute to identifying those cell wall features that have played pivotal roles in terrestrial adaptations [72,73,74].

### 4.2. Identification of Cell Wall Gene Families that Contribute to the Diversity Patterns of Cell Wall Gene Family Members in Plant Linkages

To reduce data dimensionality and visualize the potential relationships among plant species, I also subjected the diversity patterns of cell wall gene family members to principal component analysis (PCA). The distinct clusters of the plant species according to the diversity patterns of cell wall gene family members were sufficiently differentiated based on the first two primary components (PC1 and PC2). The PCA revealed that dicot and monocot plant species were separated by PC1, and the cluster including green algae, bryophyte, and pteridophyte species, was shifted in a positive direction on PC2, although there were a few, albeit important, irregular plant species (Figure 1B).

I further performed k-means clustering analysis and identified the cell wall gene families making significant contributions for each cluster by comparing the average value of the ratio of the family genes within cluster (Table S3). For example, PMT and CE8 were found to contribute predominantly to cluster 3, which included numerous dicot plant species, and I obtained high average values for the ratios of the PMT and CE8 members in this cluster (Figure 1C). As mentioned previously, the methyl-esterification and de-esterification of HGs are catalyzed by PMT and PME in CE8, respectively (Figure 2A) [75]. The methyl-esterification status of HG, which is mainly controlled by PME, directly affects the mechanical properties of the cell wall, and is associated with meristem establishment and patterning for emerging organ primordia in general vegetative and reproductive development [76,77,78,79]. Additionally, the methyl-esterification status of HGs has been shown to play multiple roles in specialized tissue differentiation, including vascular development and stomatal formation [80,81,82,83]. It is conceivable that expansion of PMT and CE8 family genes in dicot plant species has uniquely contributed to a divergence in the regulation of cell wall mechanical properties in these processes. With regard to this point, it should be noted that some non-poacea plant species belonged to cluster 3 (Table S3). Additionally, the PCA also placed the non-poacea plant species and dicot plant species close to or within the same cluster (Figure 1B). The results support that the non-poacea plant species have developed cell wall features relatively similar to that of dicot plant species [84]. In future analyses, it will be important to investigate the differences in cell wall gene families between non-poacea plant species and poacea plant species in detail, and to explore the evolution processes of cell wall structure that have developed uniquely in poacea plant species.

**Figure 3:**
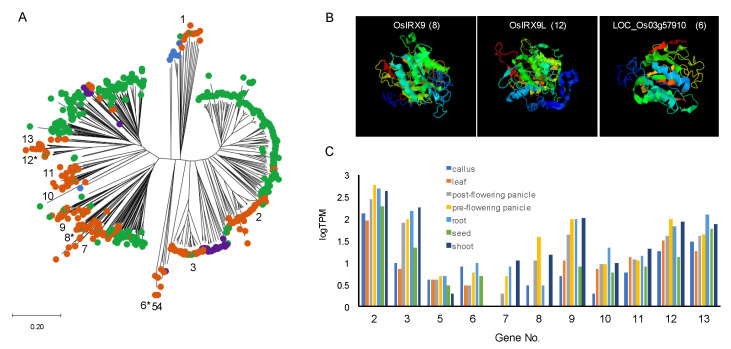
Comparative analysis of GT43 family. (**A**) Phylogenetic relationships of GT43 family among 100 plant species. Amino acid sequences were aligned by using DDBJ ClustalW 2.1 online freeware (http://clustalw.ddbj.nig.ac.jp/). Phylogenetic relationships among the proteins were constructed using the neighbor-joining method in MEGAX [94]. Each point corresponds to the member of Chlorophyta (blue), Bryophyta or Pteridophyta (purple), dicots (green), and monocots (orange). Numbers indicate the GT43 genes in *Oryza sativa*. Gene IDs are shown as bellow: 1, LOC_Os01g05400; 2, LOC_Os06g47340; 3, LOC_Os04g55670; 4, LOC_Os04g58040; 5, LOC_Os07g3999; 6, LOC_Os03g57910; 7, LOC_Os03g17850; 8, LOC_Os07g49370; 9, LOC_Os05g03174; 10, LOC_Os04g01280; 11, LOC_Os10g13810; 12, LOC_Os01g48440; 13, LOC_Os05g48600. Asterisks indicate members for homology model analysis (shown in Figure 3B). A distance scale is included at the bottom for the protein tree. (**B**) A homology model of two xylan xylosyltransferases (OsIRX9, OsIRX9L) and other type of GT43 member (LOC_Os03g57910). Structures for OsIRX9, OsIRX9L, and LOC_Os03g57910 were constructed via homology modeling with galactosylgalactosylxylosylprotein 3-beta-glucuronosyltransferase (Protein Data Bank ID: 1v82) using the I-TASSER webserver (https://zhanglab.ccmb.med.umich.edu/I-TASSER) [95]. Numbers in parenthesis correspond to those given in Figure 3A. (**C**) Expression pattern of GT43 members in *Oryza sativa*. The datasets of GT43 members in *Oryza sativa* were obtained from previously reported RNAseq data (E-MTAB-2037 from EMBL-EBI database) [96].

I also obtained high average values for the ratios of the GT43 and GT61 members in cluster 4, which includes numerous monocot plant species, and thereby identified GT43 and GT61 as predominantly contributing to cluster 4 (Figure 1C). Interestingly, both GT43 and GT61 are involved in the synthesis of (glucurono)arabinoxylans (Figure 2B). GT43 family genes encode putative xylosyltransferases required for synthesizing the xylan backbone [85,86]. Some members of the GT61 family encode putative arabinosyltransferases that mediate the α-1,3-Ara*f* substitutions of xylan [87,88]. Given that the attachment of ferulate to the arabinosyl residue leads to the formation of cross-links between the arabinoxylans via oxidative coupling of ferulic acid residues, the arabinosyl side chains of arabinoxylans is considered essential for the formation of the xylan network. Glucuronoarabinoxylan and arabinoxylan are the predominant crosslinking glycans in primary cell walls of commelinid monocotyledons, which form one of the main groups of monocot plant species, and provides a basis for the unique features of the wall architecture of these species [43,89,90,91]. The expansions of GT43 and GT61 may be associated with the development of unique cell wall structures in the commelinid monocotyledon lineage. It is speculated that an increase in gene copy number could contribute to enhancing the potential for differential expression regulation in different types of cells in the commelinid monocotyledons, and may also facilitate the acquisition of novel functions associated with the synthesis of glucuronoarabinoxylan. The GT61 family also contains a gene encoding a putative xylosyltransferase involved in mediating the xylosyl substitution of arabinosyl residues in the xylan backbone [88,92], and functional analysis of *O. sativa* GT43 has revealed the functional diversity of certain GT43 members in xylan biosynthesis [93]. Additionally, the members of GT43 show a diversity not only with respect to predicted protein structures, but also in gene expression patterns. As shown in Figure 3, phylogenetic and structural analysis reveal the presence of a variety of protein sequences in *O. sativa* and other monocot species. Analysis on the public gene expression data indicates that *OsIRX9* (LOC_Os07g49370) is predominantly expressed in shoots and flowering panicles, whereas *OsGT43E* (LOC_Os05g48600) is expressed preferentially in roots. 

It is worth noting that numerous members of the families identified both in clusters 3 and 4 play prominent roles in the formation of the cell wall network. Given that the network structure of the cell wall is the main determinant of the physical and mechanical properties of the cell wall, it is speculated that the functional divergence in these family members has significantly altered the properties of the cell wall via modification of the network structure. Additionally, increases in the copy number of these family members may have contributed to a unique development of the control of network structure dynamics in different plant species.

## 5. Concluding Remarks and Perspectives

The results presented herein indicate the evident benefit of using the available public databases for the purpose of studying multiple aspects of plant cell wall biology. The continuing rapid accumulation of sequence information provides a rich resource that enables us to collect information on cell wall genes of interest in different plant species and to perform comparative phylogenetic analyses to investigate the evolution and functional diversity of cell wall genes in plants. The diversification of plant cell wall genes has important implications for numerous interactions between plants and the surrounding environment. Owing to their sessile life histories, plants have evolved not only appropriate phenotypical adjustments in response to changing environmental conditions but also unique protective mechanisms to counter the adverse effects of environmental stress [97]. Cell walls constitute an interface for plant interactions with the environment, and consequently play multiple roles in the related molecular processes [98,99,100,101]. Numerous studies have sought to analyze cell wall dynamics in response to environmental stress, such as changes in the quality and quantity of light, submergence, dehydration, desiccation, and freezing [102,103,104,105,106]. In addition to abiotic stresses, changes in wall architecture have been widely reported in response to biotic stresses, including pathogen infection and parasitism [107,108,109,110,111]. Furthermore, the significance of cell wall dynamics has been highlighted in plant–plant interactions with parasitic plants, as well as with microbes, fungi, and insect pests [112,113]. In holoparasitic *Cuscuta* species, certain cell wall proteins involved in host-parasitic plant interactions have also been identified [114,115].

Collectively, the findings of these studies indicate that the adaptation of plants to different environmental stresses is tightly linked to the structural and functional diversity of the cell wall, which is in turn associated with the divergence in gene expression patterns and enzymatic functions. Different plant species have evolved unique cell walls as a means of adapting to different environmental stresses in diverse habitats. Given that the divergence in gene expression patterns and enzymatic functions is potentially promoted by an increase in gene copy number, the structural and functional diversity of the cell wall is considered to mirror qualitative and quantitative differences in the profiles of gene family members [61]. In this regard, the xyloglucan endotransglucosylase/hydrolase (XTH) family, one of the largest cell wall gene families, can be cited as a representative example of cell wall gene families comprising putative divergent genes [116,117]. Members of the XTH family were originally identified as enzymes catalyzing molecular grafting reactions among xyloglucan molecules or de-polymerization of xyloglucan molecules, and were considered to be involved mainly in expansive cell growth [118,119,120]. Subsequently, however, it emerged that a large number of XTH family members have divergent enzymatic functions [121,122,123,124], as well as differential patterns of gene expression in different plant species [125,126,127]. The functional and regulatory diversity of XTH members indicates that each XTH plays a particular role in modulating the wall architecture in a temporally and spatially specific manner. Molecular genetic approaches used in study of *A. thaliana* have indicated that different XTH members play particular roles in modulating cell wall structure, not only with respect to growth and development [128,129,130,131], but also in response to environmental stresses, including shade avoidance, freezing, parasitism, and aluminum sensitivity [102,106,111,132]. These studies have also indicated that numerous members of the XTH family are functionally redundant. As the acquisition of a new gene function is potentially enhanced by a relaxation of the functional constraints on redundant genes, the emergence and stability of redundant XTH genes are important with respect to potentially functional diversity, and may have additional effects on the expansion of the XTH family.

With the availability of continuously updated databases, further comparative analyses of cell wall genes will enable us to accurately characterize patterns in cell wall diversity in relation to diverse biological events, including interactions with the environment. As the growth and development of multiple types of plant cells is ultimately dependent on cell wall dynamics, it is anticipated that a more complete characterization of the functionally distinctive structure of the cell wall, combined with recent advances in technology, will make significant contributions to broad spectrum of plant sciences.

## Figures and Tables

**Table 1:** Plant cell wall gene families used in this study.

Family	Subfamily	Substrate/Product ^1^	Description ^1^
GT2			
	CesA	Cellulose	Cellulose synthase
	CslA, D	Mannan	Mannan synthase
	CslC	Xyloglucan	Xyloglucan synthase
	CslF, H, J	(1,3;1,4)-β-d-glucan	(1,3;1,4)-β-d-glucan synthase
GT8			
	GT8A	Glucuronoxylan	Glucuronoxylan glucuronosyltransferase
	GT8C	Xylan	Xylan primary oligopolysaccharide synthase
	GT8D	Xylan	Xylan primary oligopolysaccharide synthase
			Xylan galacturonosyltransferas
		HG ^2^	HG galacturonosyltransferase
GT10			Glycoprotein α-1,3-fucosyltransferase
GT14		AGP ^3^	AGP glucuronosyltransferase
GT29		AGP	AGP galactosyltransferase
GT31		AGP	AGP galactosyltransferase
GT34		Xyloglucan	Xyloglucan α-1,6-xylosyltransferase
GT37		Xyloglucan	Xyloglucan α-1,2-fucosyltransferase
		AGP	AGP α-1,2-fucosyltransferase
GT43		Xylan	Xylan xylosyltransfearse
GT47			
	GT47A	Xyloglucan	Xyloglucan β-1,2-galactosyltransferase
	GT47B	RGI ^4^	RGI arabinosyltransferase
	GT47C	Xylogalacturonan	Xylogalacturonan β-1,3-xylosyltransferase
	GT47E	Xylan	Xylan xylosyltransfearse
		Xylan	Xylan primary oligopolysaccharide synthase
GT48		Callose	Callose synthase
GT61		Arabinoxylan	Arabinoxylan α-1,3-arabinosyltransferase
GT64			
GT68			
GT77		RGII ^5^	RGII α-1,3-d-xylosyltransferase
		AGP	Arabinofuranosyltransferase
GT92		RGI	RGI galactosyltransferase
GH1		Mannan	Exo-β-1,4-mannosidase
GH3			β-glucosidase/xylosidase
GH5		Mannan	Endo-β-mannanase
GH9		Cellulose	β-1,4-glucanase
GH10		Xylan	Endo-β-xylanase
GH16		Xyloglucan	Xyloglucan endotransglucosylase/hydrolase
GH17		Callose	β-1,3-glucanase
GH18			
GH19		Cellulose	
GH28		HG	Polygalacturonase
GH31		Xyloglucan	Xyloglucan α-1,6-xylosidase
GH35		Xyloglucan	Xyloglucan β-1,2-galactosidase
GH38			
GH51		Arabinan	Bifunctionalα-L-arabinofuranosidase/β-d-xylosidase
GH95		Xyloglucan	Xyloglucan α-1,2-fucosidase
Expansin			
CE8		HG	Pectin methylesterase
CE13		RGI	Pectin acetylesterases
PL1		HG	Pectin Lyases
PL4		RGI	RGI lyses
PMT		HG	HG methyltransferase

The table is modified from [61,62]. ^1^ Substrate/Product and Description are based on the descriptions provided for characterized members of the family. ^2^ HG, homogalacturonan; ^3^ AGP, arabinogalactan protein; ^4^ RGI, rhamnogalacturonan I; ^5^ RGII, rhamnogalacturonan II.

## Supplementary Materials

The following are available online at https://www.mdpi.com/2223-7747/9/9/1195/s1, Figure S1: The total number of members of cell wall gene families in 100 species of green plants, Table S1: The ratio of the number of members of each family to the total number of protein-coding genes, Table S2: Database URL, Table S3: The clusters of plant species according to the pattern of the ratio of cell wall family genes.
