# Supplementary material for: A Genomic Perspective on the Evolutionary Diversity of the Plant Cell Wall
Source: Plants (Basel). 2020 Sep 12;9(9):1195. doi: 10.3390/plants9091195 (PMC7570368; doi:10.3390/plants9091195)
Supplement: Supplementary file 1 [file plants-09-01195-s001.zip › Figure S1.pdf]

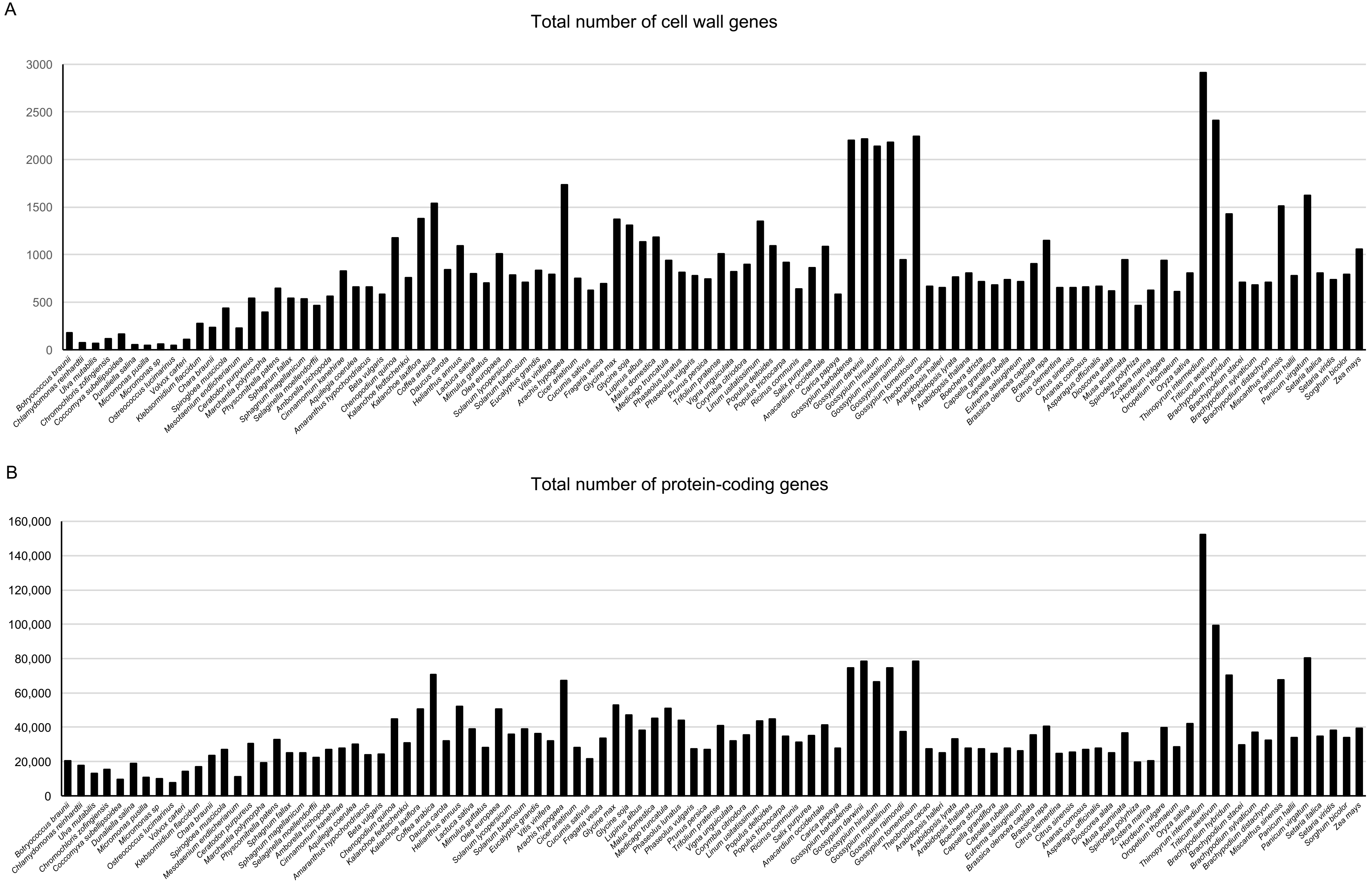


**Figure S1** The total number of members of cell wall gene families in 100 species of green plants. (A) The total number of cell wall genes of 38 cell wall gene families in 100 species of green plants. These species include 14 Chlorophyta, 5 Bryophyta, one Pteridophyta, 58 dicots and 22 monocots. The cell wall gene families consist of 16 GT families, 15 GH families, 2 PL families, 2CE families, a EXP family and a PMT familiy. (B) The total number of putative protein-coding genes in the 100 species of green plants.
